# Cerebellum-Cortical Interaction in Spatial Navigation and Its Alteration in Dementias

**DOI:** 10.3390/brainsci12050523

**Published:** 2022-04-20

**Authors:** Pierandrea Mirino, Anna Pecchinenda, Maddalena Boccia, Adriano Capirchio, Fabrizia D’Antonio, Cecilia Guariglia

**Affiliations:** 1 Department of Psychology, “Sapienza” University of Rome, 00185 Rome, Italy; pierandrea.mirino@uniroma1.it (P.M.); anna.pecchinenda@uniroma1.it (A.P.); maddalena.boccia@uniroma1.it (M.B.); 2 Ph.D. Program in Behavioral Neuroscience, “Sapienza” University of Rome, 00185 Rome, Italy; 3 Computational and Translational Neuroscience Laboratory, Institute of Cognitive Sciences and Technologies, National Research Council, 00185 Rome, Italy; adrianocapirchio86@gmail.com; 4 Cognitive and Motor Rehabilitation and Neuroimaging Unit, IRCCS Santa Lucia, 00179 Rome, Italy; 5 Department of Human Neurosciences, “Sapienza” University of Rome, 00185 Rome, Italy; fabrizia.dantonio@uniroma1.it

**Keywords:** spatial navigation, cerebellum, inferior olivary nucleus, dementia, brainstem, Alzheimer’s disease

## Abstract

The cerebellum has a homogeneous structure and performs different computational functions such as modulation/coordination of the communication between cerebral regions, and regulation/integration of sensory information. Albeit cerebellar activity is generally associated with motor functions, several recent studies link it to various cognitive functions, including spatial navigation. In addition, cerebellar activity plays a modulatory role in different cognitive domains and brain processes. Depending on the network involved, cerebellar damage results in specific functional alterations, even when no function loss might be detected. In the present review, we discuss evidence of brainstem degeneration and of a substantial reduction of neurons in nuclei connected to the inferior olivary nucleus in the early stages of Alzheimer’s disease. Based on the rich patterns of afferences from the inferior olive nucleus to the cerebellum, we argue that the subtle alterations in spatial navigation described in the early stages of dementia stem from alterations of the neuromodulatory functions of the cerebellum.

## 1. Introduction

The last decades have been characterized by an impressive body of evidence on the role of the human cerebellum in a wide range of cognitive functions, including perception, language, working memory, executive functions, behavioral control, and social cognition [1]. To date, it is well known that the cerebellum participates in complex brain networks and that its damage affects different cognitive functions. Based on the relatively uniform anatomy and physiology of the cerebellar cortex, it has been proposed that this structure performs similar computational functions in different domains [1]. Accordingly, the effect of cerebellar lesions may depend on the brain-cerebellar network involved, which may alter the network functioning rather than result in a total function loss. Among the studies linking the cerebellum to different cognitive functions, some have recently focused on the role of this structure in environmental navigation.

The present review examines the extant evidence from animal studies on the role of the cerebellum in spatial navigation. Based on this evidence, we propose that the navigational disorders observed in humans at the early stages of dementia may reflect the neurodegeneration of cerebellar structures involved in navigational cerebro-cerebellar network. Evidence in support of this interpretation comes from clinical studies, indicating that patients with different types of dementia [2,3,4,5,6] show significantly reduced navigational skills since the early stages of the disease. However, not all patients with neurodegenerative disorders show altered navigational skills. We argue that signs of navigational disorders in early stages of dementia, without or with minimal episodic memory deficits, are indicative of the involvement of cerebellar structures that are part of the navigational network in the neurodegenerative process. This account entails that the functional effects of the neurodegenerative process depend on the specific cerebellar network involved. For example, when the neurodegeneration affects connections to cerebellar structures involved in episodic memory, learning and recalling sequences of stimuli (i.e., in the Corsi test) or correctly ordering sequences of events would be altered. We review evidence indicating that early signs of spatial navigation disorders in dementia reflect connections alterations between cerebral areas and the inferior olivary complex.

The review is organized in four main sections as we (1) discuss the visuospatial skills involved in spatial navigation and their neural correlates, we provide an overview (2) of the available evidence on the cerebellar contribution to spatial navigation and (3) on the function of the inferior olivary complex in regulating the cerebellar activity. Finally, (4) we discuss the role of the cerebellum in spatial navigation disorders observed in dementia and propose that they reflect alteration of the network involving connections between the inferior olivary complex and the cerebellum.

## 2. Visuospatial Skills and Spatial Navigation

**Figure 1:**
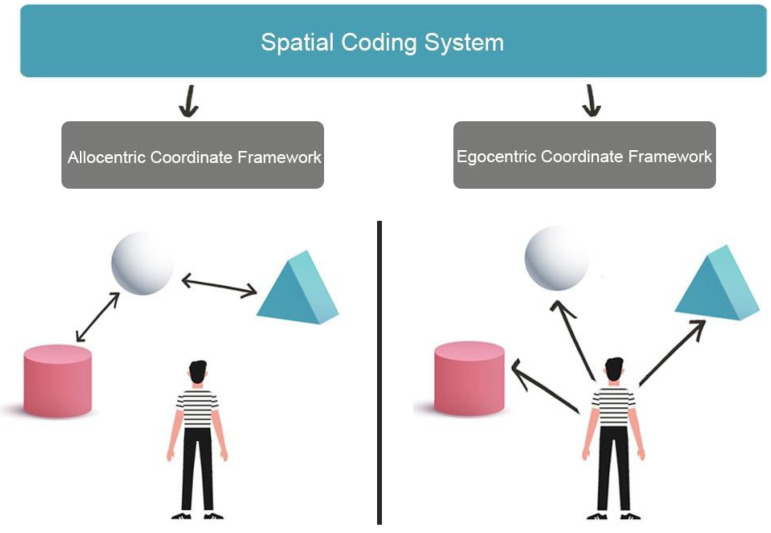
The image shows two different formats of environments representation. Allocentric Coordinate Framework encoding spatial information based on the navigator’s perception of relative landmark positions (**left**). The Egocentric Coordinate Framework bases spatial representations from the point of view of the navigator (**right**).

Spatial navigation represents the individuals’ ability to acquire new environmental knowledge, develop cognitive maps, and determine the correct path to reach a goal using multiple cues, landmarks and beacons [7]. To perform accurate spatial navigation, individuals must identify their current position and destination in the environment and trace the best path between the starting position and the destination. These activities involve higher cognitive processes, including planning and problem solving [8], which are based on perceiving, integrating and interpreting the multisensory environment signals (the so-called allothetic information, including optic flow, visual and auditory inputs) as well as signals from individual movements (the so-called idiothetic information, including motor efferent copy, proprioceptive and vestibular inputs) to generate, update and transform cognitive maps of the environment [9]. Namely, successful navigation relies on the integration of allothethic and idiotehetic cues. Therefore, visuo-spatial skills represent a set of abilities involved in visual and non-verbal reasoning, allowing to identify and estimate the observer’s spatial relations with environmental objects or between different objects located in the surrounding environment. Processing spatial relationships and mental imagery, especially mental rotation, are also essential to correctly navigate the environment [10]. Moreover, spatial navigation strongly depends on the ability to perceive, act and operate on environmental representations as a function of spatial coordinates [11]. In fact, environments can be represented in two different formats: egocentric and allocentric coordinate frameworks (Figure 1). In the egocentric coordinate framework, the position of each environmental object is coded based on the observer’s position (i.e., on the right, in front, etc.). The egocentric representation requires that idiothetic and allothetic information is dynamically updated during spatial navigation. In contrast, in the allocentric coordinate framework, each environmental object is related to the position of the spatial cues (i.e., cardinal points), static objects or landmarks located in the environment (for example with respect to the sea) [12,13] to get a spatial representation of the environment independent from the observer’s point of view.

There are several models of spatial navigation that acknowledge the role of visuo-spatial skills and specify the neural circuits involved. For instance, Byrne et al. (2007) [14], in the BBB model–so called after the three authors initials–propose that in everyday life almost all navigation tasks require a translation from the egocentric to the allocentric representation and vice versa. This suggests that most navigational tasks likely involve a mix of the two forms of representations. According to the BBB model, the neural bases for egocentric representations lay in the parietal cortex, where information of the individual’s current viewpoint based on current position and bearing is represented [14]. In contrast, allocentric representations rely on the medial temporal lobe (MTL) and involve hippocampal place cells (neurons that code specific spatial locations), providing some degree of metric knowledge about locations in the environment [15]. Finally, the retrosplenial cortex performs translation between egocentric and allocentric coordinate frames and allows aligning the current “map” in the medial temporal lobe with the current viewpoint in the parietal cortex [14].

Cognitive models also acknowledge the role of visuo-spatial skills in navigation. For instance, Siegel and White (1975) [16] propose a hierarchical developmental model of human navigation, based on three different navigational processes: (a) Landmark navigation, which refers to a process that allows individuals to reach an environmental goal by navigating toward a visible landmark in the environment; (b) Route navigation, which allows individuals to reach their goal based on the egocentric representations of the sequence of landmarks in the environment and the directions that link a landmark to the following one; (c) Survey navigation relies on the development of an allocentric cognitive map and it is characterised by configurational-gestalt elements. It gives something more than a minimal map and provides an advantage in way-finding and in organising the navigational experience [16]. A similar model was proposed by Montello [17] who suggest that route and survey representations are alternatively used according to the navigational task request. Similarly, Wang and Sperke [18], argued that three cognitive systems underlie spatial navigation in mammals: (1) the path integration system that, based on the processing of idiothetic information on movements in the environment, operates a dynamic update of the individual position, maintaining a vector between the starting position and the actual position; (2) the view-dependent recognition system involved in recognizing places, by comparing current perceptions of the surrounding environment with the images of the same place in the same perspective stored in the long-term memory; (3) the reorientation system that, allows the individual to reorient in the environment by means of a cognitive map [18,19]. In humans, other processes are also involved such as the use of language and/or of paper maps [18]. Finally, Wolbers and Hegarty [13], focus on three interdependent domains related to navigation skills: cognitive and perceptual factors, neural information processing, and variability in brain microstructure [13]. Their model predicts that even if these levels may be independent, when navigating in most natural environments, the relevant features are not detected from a single vantage point and it is essential to keep track of the individual’s position and orientation to integrate all relevant features in a complete environment representation. To sum up, existing models converge in proposing that spatial navigation relies on basic perceptual and memory processes as well as on complex multisensory processes, hence it involves wide neural networks, in which information must be integrated and manipulated in space and time [20,21]. However, none of these models points to the contribution of the cerebellum.

## 3. The Role of the Cerebellum in Spatial Navigation

Although the function of the cerebellum has typically been associated with motor functions, several recent studies point to the cerebellum being involved in various cognitive functions [22], including spatial navigation. More specifically, clinical and neuroimaging evidence [23,24] suggests a functional and anatomical distinction between sensorimotor and cognitive cerebellum. The latter includes lobule VI, Crus I and II and lobule VIIB of the posterior lobe, which have been linked to different aspects of executive functions [25].

Already in 2013, Rochefort [26] reviewing the available evidence, underlined how many studies point to the role of the cerebellum in environmental navigation, especially when considering the existing connections to the hippocampal formation. They posit that the cerebellum contributes to spatial navigation at two levels: Firstly, in processing information on autonomous movements, to build a spatial representation in the hippocampus at the level of the place cells. Secondly, in the use of this spatial representation to perform an optimal trajectory towards a goal destination. Hence they propose that the cerebellum takes part in the navigation system by shaping the firing of the place cells in the hippocampus [27]. This contribution could occur either through direct projections to the hippocampus or via multi-synaptic connections involving a thalamic relay to the posterior parietal cortex or to the retrosplenial cortex.

An important contribution also comes from a recent study [28] showing that the cerebellum contributes to visuospatial working memory and decision-making, by optimizing the task-related modulation of mPFC-hippocampus (dCA1) gamma coherence. Findings about neural recording during both cognitive and sensorimotor processing strongly suggest that the cerebellum has a pivotal role in coordinating communication within network modulating the frequency coherence of cerebral cortical areas. Such a role is consistent with the long recognized role of the cerebellum in timing and temporal coordination [28]. Taken together, this evidence suggests that the cerebellum assumes a modulatory role in each process in which it is involved, being it motor or cognitive processes. Indeed, this proposal together with the cortico-nuclear micro-complex architecture of the cerebellum [29] inspired the universal cerebellar transform (UTC), a model describing the cerebellum activity as an unique, universal type of computation [30]. In other words, according to these models, all the cerebellar areas work in similar ways, by providing the same type of input and playing the same role in all the networks in which the cerebellum is involved. In the UTC model, the cerebellum integrates internal representations with external stimuli, modulating the different information flows that underlie a wide range of functional domains, optimizing individual’s performance based on context [31].

Importantly, the model predicts that lesions in different cerebellum areas should have similar effect on different domains depending on the cerebrellum-brain network affected by the lesion (Universal Cerebellar Impairment-UCI) [31,32]. Indeed, there is some evidence that this is the case as in humans, cerebellar lesions can result in different cognitive deficits [33]. This led some authors to suggest that alterations in sequential processing is the common denominator of the cerebellum contribution to cognitive, affective and behavioral deficits [34]. However, animal studies show that cerebellectomized rats exhibit different exploration patterns of a novel environment, suggesting that cerebellum is involved in processing spatial information and elaborating motor strategies during exploration [35]. Specifically, the growing evidence on the involvement of the cerebellum in navigation has raised the question of the potential roles of the two major cerebellar inputs, the olivo-cerebellar input (climbing fiber; CF) and the pontine nuclei-granule cells-parallel fibers input (mossy fiber; MF). Indeed, lesion studies provide some evidence that cerebellar dysfunctions may depend, at least in part, on alterations of the olivo-cerebellar inputs. For instance, Rondi-Reig [36] tested rats with lesion of CF and/or MF of the cerebellum in either the cued or the place protocol of the water maze. Rats with CF lesions and partial or total MF lesions presented a deficit in the latency to find a hidden platform in the water maze but were still able to find it when visual cues were available.

### Cerebellum as Part of a Complex Network

**Figure 2:**
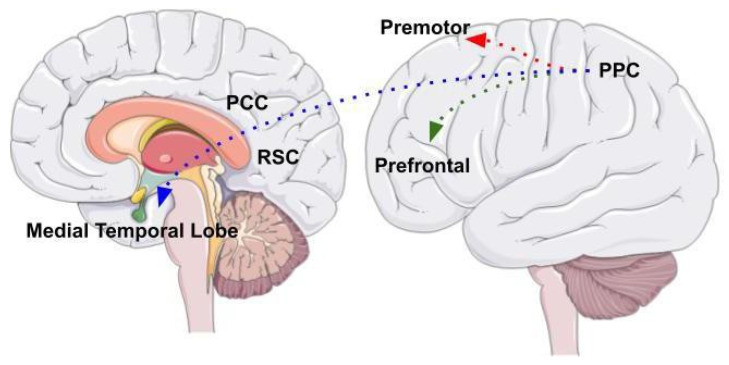
The image shows the three different neural circuits originating from PPC areas. The parieto-prefrontal pathway, the parieto-premotor pathway, and the parieto-medial temporal pathway. The figure was drawn based from Kravitz et al., (2021).

The neural system underlying visuospatial abilities and spatial navigation includes the posterior parietal cortex (PPC) as a central area that is part of a complex, wide network of different brain areas (Figure 2). The posterior parietal cortex receives visual, auditory, and vestibular information and integrates different sources of information in a complex representation of the environment. Three different neural circuits originate from different posterior parietal cortex areas [37].

One is the parieto-prefrontal pathway, which involves lateral intraparietal area (LIP), ventral intraparietal area (VIP), medial temporal area (MT) and medial superior temporal area (MST) that project to the prefrontal cortex. This pathway contributes to the visual processing of information and spatial working memory [38]. The other is the parieto-premotor pathway, which includes two different parallel projections that connect the PPC to the premotor areas. The first projection connects the parietal reach region (PRR) to the dorsal premotor cortex whereas the second projection connects VIP with the ventral motor cortex. Overall, the parieto-premotor pathway mediates eye moments, reaching, grasping and other forms of visually guided actions [37]. Finally, the parieto-medial temporal pathway connects the caudal region of the inferior parietal lobe (cIPL) to several areas located in the medial temporal lobe (MTL). This pathway is involved in different spatial functions related to spatial orientation [37]. The cIPL processes the egocentric information and sends efferences to different MTL areas: the posterior cingulate cortex (PCC), that participates in directing and shifting attention; the retrosplenial cortex that is involved in memory, imagery and planning processes and translating representations from the egocentric to the allocentric format and vice versa; the parahippocampal gyrus, that contributes to the allocentric representation of the environment; the hippocampus that provides a crucial contribution in spatial navigation but whose role has not yet been fully clarified [38].

The neural architecture involving connections to the posterior parietal cortex is acknowledged by the BBB model [14], according to which the posterior parietal cortex underlies processing the spatial visual information and represents the environment in an egocentric coordinate framework. Subsequently, the MTL areas receive the egocentric representation through the parieto-medial temporal pathway and integrate it with the allocentric representation elaborated by the parahippocampal gyrus. Finally, the retrosplenial cortex converts egocentric in allocentric representations and vice versa, in a bidirectional way, while the hippocampus allows for the long-term storage of these representations [14]. However, this architecture does not account for evidence indicating that deficits in navigation may result from cerebellar alterations, which affect brain areas to which the cerebellum is connected, therefore mimicking the impairments generated by direct alterations of frontal and parietal areas.

More specifically, studies using viral tracers in nonhuman primates show a di-synaptic projection from the dentate nucleus (DN) towards the prefrontal cortex, passing through the median-dorsal and ventrolateral nuclei of the thalamus [26]. This suggests the presence of a circuit connecting the lateral part of the cerebellar hemispheres and the dentate nucleus with the dorsal region of the prefrontal cortex [39]. Animal studies also suggest that in mice this thalamic projection supports the prefrontal activity during the maintenance of spatial working memory [40]. These prefrontal areas are involved in executive functions, such as strategic behavior, planning, abstraction, cognitive flexibility, and working memory, all involved in spatial navigation. On the other hand, the prefrontal cortex areas send rich projections to the pontine nuclei, from which the mossy fibers (MF) originate. Importantly, these projections constitute one of the two main inputs of the lateral cerebellar hemispheres [39,41,42,43]. Bidirectional connections between the cerebral cortex and the cerebellum form the cerebro-cerebellar circuit, which represents the anatomical substrate for the cerebellar influence on higher cognitive functions [44]. In addition, MRI studies show robust functional connectivity between the dentate nucleus–the major cerebellar output nucleus–and several cortical and subcortical cerebral areas involved in spatial navigation, including the parahippocampal gyrus and the hippocampus, but also parietal, frontal areas, thalamus and insular cortex [45].

Clinical studies also provide some converging evidence. For example, Tedesco et al. [46] tested navigational working memory in 12 cerebellar patients and 12 healthy age-matched participants using 2 comparable navigational tests (Walking Corsi Test and the Magic Carpet) [2,47]. The Walking Corsi Test (i.e., WalCT) [9,48] includes nine black tiles (30 cm × 30 cm) that are placed on a light gray carpet (2.50 m × 3 m). At the edge of the carpet, a 10th tile shows the participant’s position. The examiner presents sequences of increasing length by walking on the carpet and stopping on each tile for 2 s. After the presentation of each sequence, the participant is required to walk on the carpet and repeat the sequence. The Magic Carpet (i.e., E-WalCT) [47,49], has the same dimensions as the WalCT (2.50 m × 3 m), with nine white tiles (30 cm × 30 cm) placed on the carpet in the same array. Each tile is 10-mm thick with a luminous white surface of 75 mm × 95 mm on top and 6 pressure sensors that are regularly spaced under the surface. As in the WalCT, at the edge of the carpet, a tenth tile shows the participant’s position. The tiles are connected to an electronic device that turns the luminous surface on or off and detects the sensors activated by walking on the tiles. Sequences of increasing length of lighting tiles are presented. At the end of each sequence, all the lights go out and an acoustic warning is the participant’s signal to reproduce the observed sequence by walking on the E-WalCT. Patients with cerebellar lesions performed significantly worse than control participants only at the E-WaLCT. This finding has been attributed to the fact that the E-WalCT involves a higher cognitive load to detect and order single, independent stimuli as a sequence and that this type of processing is highly dependent on the contribution of cerebellum. In fact, Perrochon [47] suggest that spatial mapping requires a translation from stimulus space onto response space and this translation entails higher processing costs in the E-WalCT than in the WalCT. Accordingly, impaired cerebellar function affects stimulus-response associations in working memory, preventing sequence elaboration. It should be noted that this account is in keep with the theory proposed by Spencer [50], according to which the cerebellum works with cortical regions “to sustain representations of the stimulus-response mappings, a form of action-based working memory” (pp. 1302).

To sum up, that the cerebellum plays an important role in many cognitive, sensorimotor, and behavioral functions is well accepted. In contrast, the role of the cerebellum in human navigation has been less explored despite evidence from animals’ studies [36,51] consistently showing its key role in developing, storing, and retrieving cognitive maps of the environment. For instance, data suggest that it plays an important role in allowing to put in the correct sequence a set of spatial position [46] but it is still unclear the type of inputs that allows the cerebellum to play its role in human navigation. Given the functional anatomy of cerebellum, the input from the inferior olivar complex seems a possible candidate as it plays an important role in modulating the cerebellar activity and it could be involved in coding temporal/sequential features of different types of stimuli [52].

## 4. The Inferior Olivary Complex: A Complex Hub in the Brainstem to Regulate the Cerebellar Activity

The inferior olive (IO), together with the pontine nuclei, represents the primary afferences to the cerebellum. Alterations in the activity of the inferior olive should result in alterations of cerebellar activity and consequently, of the activity in the cerebral areas to which the cerebellum is connected (and whose activities are modulated by cerebellum), including those responsible for the cognitive processes of spatial navigation.

The inferior olivary complex (IO) is an ovoidal grey matter region located in the medulla oblongata, the ventral part of the brainstem. Its anatomical position is near the lateral posterior furrow, where the hypoglossal, glossopharyngeal, vagus and accessory nerves emerge [53]. IO forms two visible external protrusions on the medulla oblongata bilaterally to the bulbar pyramids. Each protrusion consists of three subunits: the principal olive, the medial accessory olive, the dorsal accessory olive. The medial and the dorsal accessory olive are also named “para-olive” [54]. In the human brain, the IO is mainly constituted (85%) by the principal olive (PO), whereas in monkeys and rodents, the largest nucleus is the medial accessory olive. The IO has the highest density of electrical synapses found in the adult human brain and provides the most substantial input to the cerebellum, sending glutamatergic projections, named climbing fibers (CFs), to Purkinje cells (PCs) [55].

Two main types of neurons are found in the principal olive: the large multipolar excitatory neurons (90% of the PO cells) and the small inhibitory interneurons (10% of the PO cells). The large multipolar excitatory neurons are glutamatergic cells with long axons, that extend rostrally from the nucleus to form the climbing fibers that, in turn, project to the contralateral cerebellar cortex. Furthermore, these axons send collateral fibers to the dentate nucleus. Principal olive neurons act as an electrical syncytium due to the presence of gap-junctions, which constitute dendro-dendritic electrical synapses. Each principal olive neuron is electrically coupled with about fifty neighboring neurons and discharges rhythmically and synchronically with them [56,57].

**Figure 3:**
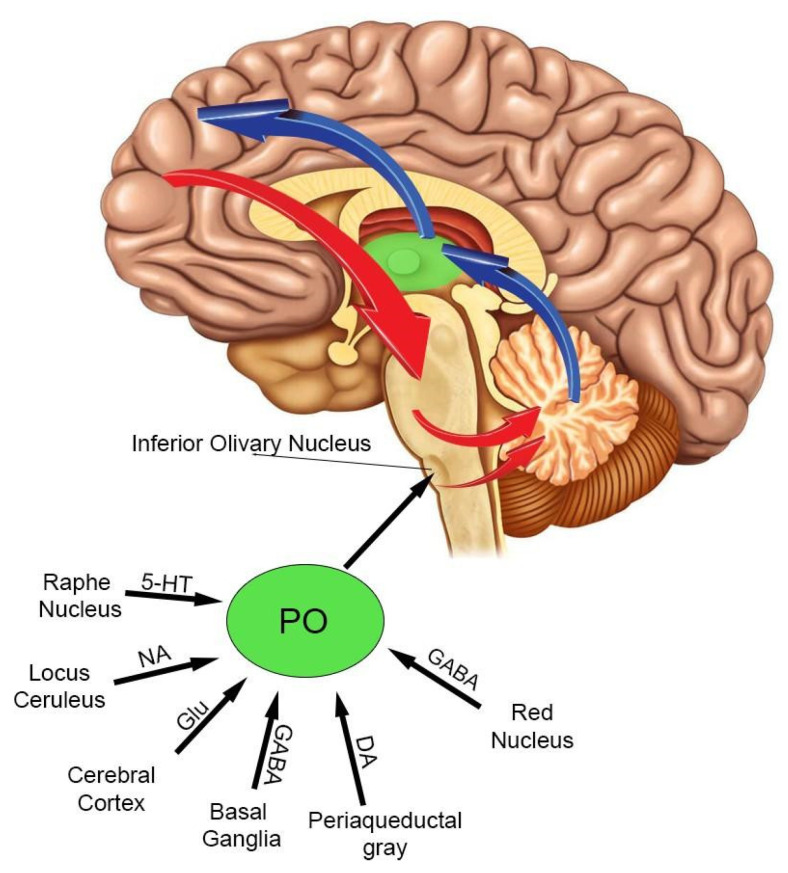
From the inferior olive nucleus originate the climbing fibers that go to the cerebellum, making synapses with the cells of the Purkinje, and to the dentate nucleus. The dentate nucleus, modulated by the activity of this interaction (olivo-Purkinje), projects, through the thalamus, to the prefrontal cortex which in turn projects to the pontine nuclei from which the mossy fibers for the cerebellum depart, thus closing this ring that we could call olivo-cerebello-cortico-pontine loop (oCCP).

The principal olive receives projections from different brain areas (Figure 3). The glutamatergic excitatory projections mainly originate from the contralateral and ipsilateral motor cortex [58,59]. The serotonin (5HT) projections start in the raphe and strongly relate to the rhythmicity of the principal olive [60]. The dopaminergic projections originate in the periaqueductal grey matter [61], while noradrenergic projections start from the medulla oblongata but, curiously, not from the locus coeruleus [62] Indeed, the locus coeruleus is considered a homogenous nucleus, involved in learning [63], from which noradrenaline (NE) is released in a diffuse way to forebrain, brainstem, cerebellum, and spinal cord [64]. In human IO the noradrenaline fibers are more homogeneously distributed than they are in rat, cat, and monkey IOs [62,65].

The prominent inhibitory projections (GABAergic) to the principal olive start from the parvocellular part of the red nucleus, basal ganglia and dentate nucleus (DN) [61,66,67,68]. The parvocellular part of the red nucleus terminates in the ipsilateral principal olive connecting the olivo-cerebellar system with the motor and premotor cortices [69].

The outputs of the principal olive are the climbing fibers (CFs). These fibers pass into the restiform body projecting to the cerebellar cortex and, concurrently with the mossy fibers (MFs), represent the principal inputs to the cerebellar cortex, very likely playing a pivotal role in modulating cerebellar activity. A single climbing fiber carries impulses at frequencies lower than 10 Hz and innerves a single adult Purkinje cell (topographical relation 1:1) with multiple excitatory synapses. CFs axon terminals wrap around the Purkinje cells body and proximal dendrites in multiple points [70,71]. A climbing fiber causes a strong depolarization of the Purkinje cell, which triggers a short train of spike bursts (the ”complex spike”), and can induce long term depression (LTD) [71]. The dentate nucleus (DN) operates the most vigorous control over principal olive synchronization [67,72]. It is noteworthy that the dentate nucleus output depends on the balance between the excitatory and the inhibitory signals received by the Purkinje cells. Therefore, the IO signals modulate the output of dentate nucleus, closing a circular loop between IO and cerebellar cortex. Accordingly, the IO represents the hub from which different brain areas integrate their signals to modulate the principal olive and cerebellar cortex activities.

**Figure 4:**
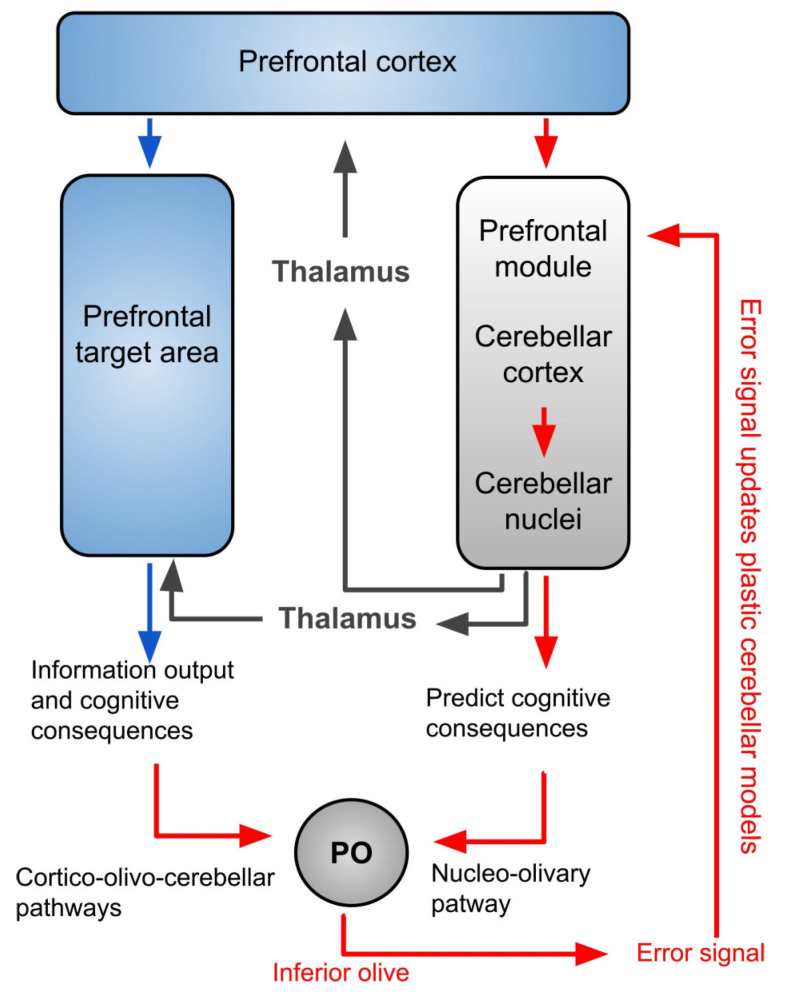
Cerebellar model of processing in prefrontal targets, extension of the forward cerebellar cortical model published by Ramnani (2006). This model is involved in the simulation of information processing in which the cerebellar cortical circuit simulates the processing of information in the targets of all areas that project to the cerebellum through the cortico-ponto-cerebellar system. The information arising in the prefrontal cortex is copied to the cerebellum. In this scheme, cerebellar forward models mimic the input–output relationships of prefrontal targets. Forward models might therefore be able to mimic information processing that is intrinsic to the prefrontal cortex.

Based on the motor cerebellar-cortical forward computational model (Figure 4) that specifically simulate motor information processing, Ramnani [73] describes a cortico-cerebellar loop shared by all cerebro-cerebellar networks. In this loop, inputs from the cerebral cortex are sent to the IO through the cortico-olivo-cerebellar pathway and from cerebellar nuclei though nucleo-olivary pathway. This organization suggests that the inferior olive could serve as a comparator for these two signals and sends “error signals” when the information received does not match. Therefore, the IO could be the principal structure involved in processing information in the cortico-cerebellar loop.

Based on the architectural connections of the IO, several studies suggest that IO is involved in the ability to detect stimuli synchronicity that lay at the basis of spatial navigation functions [52,74,75]. These studies demonstrate that the IO nucleus is sensitive to sensory input about the temporal structure of stimuli regardless of their nature, and that IO activity is not affected by the subjects’ performance, motor coordination, or effort. In fact, Teghil [76] showed two partially segregate neurocognitive systems, that process different types of time information and rely on different neural networks: the Internal-Based Timing (IBT) process, that among other areas includes the inferior olivary nucleus, mainly involved in processing idiothetic inputs, and the External-Cued Timing (ECT) process, mainly involved in processing allothetic information. IBT should play a specific role in acquiring environmental information during the exploration of novel environment, allowing to correctly code the sequence of landmarks and directions, as well as in monitoring the navigation in familiar environments, making the individual aware of having mistaken the route when an expected landmark is not met after the expected travelling time.

Similarly, Ito [77], suggests that in the early stages of learning, control signals arising in prefrontal areas act on representations in temporal and posterior parietal association areas. Through learning, these representations are effectively copied to the cerebellum, and the prefrontal cortex then acts following the rules of cerebellar forward models [77]. Moreover, in a recent model built with spiking neurons, Caligiore and Mirino [78] show how the cerebellum and the medial prefrontal cortex strongly cooperate in associative learning, assisting the work of specific areas for the proper functioning of the system. Finally, forward models support the existence of two systems working in parallel, one learning from the other and simulating its operations. In these models, the IO compares the activities of the two systems and sends a feedback error signal to the prefrontal or motor modules if the two representations are not identical, for updating plastic cerebellar models. An impairment at this level will cause dysfunctions of the updating necessary for the correct processing of information and actions.

Evidence about the involvement of olive-cerebellar system activity in the spatial navigation also comes from lesion studies on animals. Following unilateral transection of the inferior cerebellar peduncle (pedunculotomy), which unilaterally interrupts the olivo-cerebellar pathway, reinnervation occurs from the remaining peduncle towards the denervated hemi-cerebellum in new-born rats, but not in animals who received the pedunculotomy 11 days from birth, that, due to this lack of reinnervation, show severe deficits in spatial navigation tests, such as the water maze. Compared to controls, rats with later pedunculotomy do not show spatial learning and even after several trials are not able to perform direct journeys to reach the platform. In contrast, animals with pedunculotomy at birth, that have completed the reinnervation process, are able to learn the position of the platform, although less effectively than controls, and show difficulties in maintaining memory from one day to the next [79,80,81].

To sum up, there is evidence that the IO plays a crucial role in monitoring and modulating the cerebellar cortical circuit. Based on this evidence a possible specific role of IO in processing the timing components of navigational inputs has been suggested. Next, we are discussing the early signs of navigation deficits observed in different types of dementia as due to the likely involvement of the IO in the neurodegeneration of the cerebellar cortical circuit.

## 5. Cerebellum and Spatial Navigation Disorders Involved in Dementia

In the early stages of the disease, patients with different types of dementia–Alzheimer’s disease [2,3,4,5,6], Lewy body dementia [82], and vascular dementia [83]–may show signs of topographical disorientation (TD) and reduced navigation skills [2], which worsen as the neurodegenerative process proceeds [84].

According to Aguirre and D’Esposito [85] topographical disorientation includes four different types of deficits: (a) egocentric disorientation, that is the inability to represent the position of the environmental objects with regard to self; (b) anterograde disorientation, that is difficulties in learning and retrieving novel environments; (c) heading disorientation, that is the inability to associate directional information with landmarks; (d) landmark agnosia, that is the inability to recognize salient aspects of the surrounding environment and use them to orient and direct navigation.

Egocentric disorientation and heading disorientation seem to characterize the episodes of topographical disorientation that are described in dementia, sometimes as the very first symptom. However, some recent studies underline the possibility that these deficits are preceded by anterograde disorientation. More specifically, Bianchini [2] report an egocentric deficit of topographic working memory (WM) in the early stages of Alzheimer’s disease (AD). They found that navigational working memory was severely damaged, despite other types of WM were still unaffected. The result that topographic WM is selectively impaired, while verbal WM and visuo-spatial WM for reaching space are still within normal range, suggests a greater involvement of this type of memory compared to others in the early stages of Alzheimer’s disease, even when clear signs of navigational deficits have not been detected. Interestingly, there is evidence that topographic learning is also impaired in preclinical stages of Alzheimer’s disease, not only being the deficit more severe than that observed in reaching space, but also being topographical memory deficits present also in individuals who did not show any impairment in spatial learning in reaching space [86]. Additional evidence [87] also points to a higher specificity of spatial navigation and orientation deficits compared to episodic memory deficits, in differentiating Alzheimer’s disease from other dementias, especially from frontotemporal dementia. Indeed, evidence from animal studies shows that Alzheimer’s disease pathophysiology in brain areas specifically involved in navigation well before areas involved in episodic memory [87,88]. Therefore, it is not surprising that in 2018, Coughlan and colleagues [89] pointed out the great potential of using spatial navigation and orientation performance as diagnostic measures and predictors of Alzheimer’s disease pathophysiology.

Indeed, Alzheimer’s disease neuropathology, both at the early or preclinical and asymptomatic stages, is strongly related to brain atrophy in areas that are part of the spatial navigation network, including the medial temporal lobe and more specifically the entorhinal cortex, perirhinal cortex and hippocampus [90]. This entails that, early navigational alterations are likely due to neurodegeneration of the retrosplenial and entortinal areas. Since the neurodegeneration observed at the early stages of Alzheimer’s disease (Braaks stage I-II) does not seem to directly affect the hippocampus but the entorhinal cortex, that is part of the navigation network [91], this explains why navigational memory disorders would appear well before episodic memory disorders. In fact, difficulties in recognizing places and retrieving routes, are often reported as the very early symptoms by patients with Alzheimer’s disease [92]. It is also possible that in some individuals the neurodegeneration expands towards retrosplenial areas, and the early involvement of retrosplenial cortex in Alzheimer’s pathology plays an important role in the development of navigational disorders. For instance, Boccia et al. (2016) [93], in an experimental navigation task during an fMRI session with eight patients diagnosed with aMCI and eight age-matched control participants, showed that the deficit in navigational abilities and the resulting topographical disorientation in aMCIs are related to hypoactivation of the structures beyond hippocampal formation, such as the parahippocampal gyrus (PHG) and retrosplenial cortex (RSC). Importantly, both the parahippocampal gyrus and retrosplenial cortex are crucial in supporting topographical memories about new and old environments [93]. In addition, early neurodegeneration of other regions can also be involved, since not all individuals affected by Alzheimer’s disease experience navigational disorders and topographical disorientation at the early stages. Moreover, in animal studies, navigational deficits are also described following lesions in areas different from the hippocampus. Several animal studies have shown that cerebellar damages affect navigational memory [94] other than navigational planning and strategies [51,95]. A study on navigational skills and navigational memory in mice revealed a widespread network centered around the cerebral cortex and basal ganglia during the exploration of an arena, as well as a network dominated by hippocampal and cerebellar activity, which sustains sequence-based, goal-directed navigation but also contributes to the dynamics of acquisition of explorative behaviour [96]. Therefore, it is possible that patients affected by Alzheimer’s disease may show, as a very early symptom, episodes of topographical disorientation when the neurodegeneration involves the cerebro-cerebellar network involved in navigation.

Furthermore, a resting state functional magnetic resonance imaging (fMRI) study [97] provides the first evidence of altered functional connectivity between the dentate nucleus and specific cerebral structures in many regions of the temporal lobe, that are the most affected in Alzheimer’s disease [98]. The neurodegeneration of this region is considered responsible for episodic memory deficits commonly associated with right temporal lobe atrophy [99], as well as with alterations in spatial cognition [100]. Importantly, there is also evidence that cortical cerebellar outputs direct to the DN, which projects neural fibers to the cerebral cortex [101]. Finally, neuroimaging evidence shows cerebellar atrophy in Alzheimer’s disease [102,103] involves the posterior lobes (lobules VI and Crus I-II) [104], known to have strong functional connectivity with distinct associative cerebral regions [105]. Therefore, it is very likely that the cerebellum-cortical circuits are affected by the main degenerative disorders at the very early stages of the pathology. It is also likely that the involvement of this circuit extends to other neighbouring, cerebellar areas affecting cerebellar-cortical connections.

As a final remark, it is also worth noting some similarity between symptoms shown by patients with dementia and symptoms shown by patients with Cognitive Cerebellar Affective Syndrome (CCAS), a pathological condition that can be referred to as a constellation of deficits in the cognitive domains of executive function, spatial cognition, language, and affective regulation, resulting from damage to the cerebellum [106,107]. In fact, both cases are characterized by early onset of behavioural and emotional disturbances, that may occur well before episodic memory disorders, such as subtle changes in the sleep/wake rhythm, anxiety/depression phenomena and behavioural alterations. Based on the findings by Wilson and coll. [79], it is well possible that episodes of topographical disorientation and navigation alterations in the initial stages of Alzheimer’s disease, directly or indirectly, involve the olivo-cerebellar system.

To sum up, our hypothesis is that, a cerebellum dysfunction due to the neurodegenerative pathology in Alzheimer’s disease may be partially responsible for the early appearance of emotional and affective disorders, caused by functional disconnections due to the malfunctioning of some nodal nuclei. Based on the evidence above discussed, we argue that among the cerebellar nuclei, the inferior olive, which plays a primary role in the updating of plastic cerebellar models [73] can be identified as the most likely candidate. Indeed, even a partial degeneration of IO should yield difficulties in effectively acting as a node of communication between cortical cortices and the cerebellum. This in turn would produce a disconnective disorder, and a deficit in processing timing, affecting the ability to compute metrical aspect (i.e., distances) of environmental navigation.

## 6. Conclusions

Physiological and neuroimaging studies show a high correlation between the olivo-cerebellar system activity, the brainstem structures, and the cortical areas responsible for visuospatial functions. The interaction among these neural structures is responsible for the dynamicity of updating processes in models of motor activity and cognitive functions over time. This function is essential for processes that operates a continuous update of the individual position in the environment–like path integration–mainly based on processing idiothetic information about an individual’s movements in the environment. It is also very important for processing the metric features of the environment, since by processing times, it contributes to the evaluation of distance between different points of the environment. Thus, the olivo-cerebellar system and the connected cortical circuits may represent an essential hub involved in communication between different brain areas during the acquisition and the retrieval of environmental information for navigational purposes.

Indeed, some studies point to the primary role of the inferior olive-cerebellar system in coding temporal information, regardless of motor behaviour. Temporal coding plays an important role in learning and representing environments, especially complex ones, as well as in planning a route and monitoring the correctness of navigation. It is possible that the role played by olive-cerebellar system in navigation consists in processing the temporal components of environmental representation and navigation. Therefore, the altered processing of temporal information attributable to a dysfunction of this system should correspond to altered ability to learn environments (affecting the ability to learn which landmark was met before and which was meet after along a route) as well the ability to monitor navigation, making individuals not able to compute distance in relation to the speed of their translations. Measuring the relation between time processing and visuospatial abilities could allow to better understand navigational processes and the underling neural network, allowing also to develop novel ways for early detection of preclinical signs of neurodegenerative disorders.

## Data Availability

Not applicable.

## Supplementary Materials

The following are available online at https://www.mdpi.com/article/10.3390/brainsci12050523/s1, Supplementary Material A: Selection Criteria for Paper.

## Selection Criteria

The PubMed database was searched for papers published in English between 2000 and 2022 using the following keywords and their possible combinations: “cerebellum”, “inferior olivary nucleus”, “dementia”, “alzheimer” and using the following exclusions: NOT ((parkinson) OR (eeg) OR (meg) OR (psychiatric) OR (tremor) OR (ataxia)) AND ((sequen *) OR (time) OR (time) OR (processing) OR (navigation *) OR (learning) OR (cognitive *) OR (spatial) OR (orientation) OR (memory)). The complete strings used to perform the search on PubMed are provided in Supplementary Material.
