# Supplementary material for: Cerebellum-Cortical Interaction in Spatial Navigation and Its Alteration in Dementias
Source: Brain Sci. 2022 Apr 20;12(5):523. doi: 10.3390/brainsci12050523 (PMC9138670; doi:10.3390/brainsci12050523)
Supplement: Supplementary file 1 [file brainsci-12-00523-s001.zip › brainsci-1680665-supplementary.pdf]

Supplementary Material A: Selection Criteria for Paper

((Cerebellum) OR (inferior oliv*) OR (olivocerebellar system)) AND ((timing) OR (time) OR (prediction) OR (working memory) OR (processing) OR (spatial navigation) OR (dementia) OR (alzheimer) OR (sequence) OR (cognition) OR (spatial learning) OR (orienting) OR (prediction) OR (expectation)) AND (2000:2022[pdat])

(inferior oliv*[Title]) AND (Alzheimer)

((inferior oliv*[Title])) AND ((dementia) OR (Alzheimer))

(cerebellum[Title]) AND ((Alzheimer) OR (dement*))

(cerebellum[Title]) AND ((Alzheimer) OR (dement*)) AND (2000:2022[pdat])

((inferior oliv*[Title]) OR (cerebellum[Title])) AND ((dementia) OR (Alzheimer)) AND (2000:2022[pdat])

((inferior olive[Title]) OR (olivo*[Title])) AND ((dementia) OR (Alzheimer)) AND (2000:2022[pdat]) 1 (Olivocochlear)

((inferior olive[Title]) OR (olivo*[Title]) OR (cerebellum[Title])) AND ((dementia) OR (Alzheimer))

((inferior olive[Title]) OR (olivo*[Title]) OR (cerebellum[Title])) AND ((dementia) OR (Alzheimer)) AND (2000:2022[pdat])

((Cerebellum) OR (inferior olive nucleus) OR (olivocerebellar system)) AND

((timing) OR (time) OR (prediction) OR (working memory) OR (processing) OR (spatial navigation) OR

(dementia) OR (alzheimer) OR (sequence) OR (cognition) OR (spatial learning) OR (associative learning) OR (orienting) OR (prediction) OR (expectation))

NOT ((parkinson) OR (eeg) OR (meg) OR (psychiatric) OR (tremor) OR (ataxia)) AND ((sequen *) OR (time) OR (time) OR (processing) OR (navigation *) OR (learning) OR (cognitive *) OR (spatial) OR (orientation) OR (memory)).

In a period from 2000 to 2022, using the following principal keywords and their possible combinations:

((cerebellum [Title]) OR (inferior oliv * [Title])) AND ((dementia) OR (alzheimer)).

((cerebellum[Title]) OR (inferior oliv*[Title]))

AND ((dementia) OR (alzheimer))

NOT ((parkinson) OR (eeg) OR (meg) OR (psychiatric))

AND ((sequen*) OR (timing) OR (time) OR (processing) OR (navigation*) OR (learning) OR (cognit*) OR (spatial) OR (orienting) OR (memory))

AND (2000:2022[pdat])

((cerebellum[Title]) OR (inferior oliv*[Title]))

AND ((dementia) OR (alzheimer))

NOT ((parkinson) OR (eeg) OR (meg) OR (psychiatric) OR (tremor) OR (ataxia))

AND ((sequen*) OR (timing) OR (time) OR (processing) OR (navigation*) OR (learning) OR (cognit*) OR (spatial) OR (orienting) OR (memory))

AND (2000:2022[pdat])

((inferior oliv*[Title]))

AND ((dementia) OR (alzheimer))

NOT ((parkinson) OR (eeg) OR (meg) OR (psychiatric))

AND ((sequen*) OR (timing) OR (time) OR (processing) OR (navigation*) OR (learning) OR (cognit*) OR (spatial) OR (orienting) OR (memory))

AND (2000:2022[pdat])

((inferior oliv*[Title]))

AND ((dementia) OR (alzheimer))

NOT ((parkinson) OR (eeg) OR (meg) OR (psychiatric) OR (tremor) OR (ataxia))

AND ((sequen*) OR (timing) OR (time) OR (processing) OR (navigation*) OR (learning) OR (cognit*) OR (spatial) OR (orienting) OR (memory))

AND (2000:2022[pdat])
